# Intestinal microbial communities associated with acute enteric infections and disease recovery

**DOI:** 10.1186/s40168-015-0109-2

**Published:** 2015-09-22

**Authors:** Pallavi Singh, Tracy K. Teal, Terence L. Marsh, James M. Tiedje, Rebekah Mosci, Katherine Jernigan, Angela Zell, Duane W. Newton, Hossein Salimnia, Paul Lephart, Daniel Sundin, Walid Khalife, Robert A. Britton, James T. Rudrik, Shannon D. Manning

**Affiliations:** Department of Microbiology and Molecular Genetics, Michigan State University, East Lansing, MI 48824 USA; University of Michigan, Ann Arbor, MI 48109 USA; Wayne State University, Detroit, MI 48202 USA; Spectrum Health, Grand Rapids, MI 49503 USA; Sparrow Hospital, Lansing, MI 48912 USA; Department of Molecular Virology and Microbiology, Baylor College of Medicine, Houston, TX 77030 USA; Michigan Department of Health and Human Services, Bureau of Laboratories, Lansing, MI 48913 USA

**Keywords:** 16S rRNA, Intestinal microbiome, Enteric pathogens

## Abstract

**Background:**

The intestinal microbiome represents a complex network of microbes that are important for human health and preventing pathogen invasion. Studies that examine differences in intestinal microbial communities across individuals with and without enteric infections are useful for identifying microbes that support or impede intestinal health.

**Results:**

16S rRNA gene sequencing was conducted on stool DNA from patients with enteric infections (*n* = 200) and 75 healthy family members to identify differences in intestinal community composition. Stools from 13 patients were also examined post-infection to better understand how intestinal communities recover. Patient communities had lower species richness, evenness, and diversity versus uninfected communities, while principle coordinate analysis demonstrated close clustering of uninfected communities, but not the patient communities, irrespective of age, gender, and race. Differences in community composition between patients and family members were mostly due to variation in the abundance of phyla *Proteobacteria*, *Bacteroidetes*, and *Firmicutes*. Patient communities had significantly more *Proteobacteria* representing genus *Escherichia* relative to uninfected communities, which were dominated by *Bacteroides*. Intestinal communities from patients with bloody diarrhea clustered together in the neighbor-joining phylogeny, while communities from 13 patients’ post-infection had a significant increase in *Bacteroidetes* and *Firmicutes* and clustered together with uninfected communities.

**Conclusions:**

These data demonstrate that the intestinal communities in patients with enteric bacterial infections get altered in similar ways. Furthermore, preventing an increase in *Escherichia* abundance may be an important consideration for future prevention strategies.

**Electronic supplementary material:**

The online version of this article (doi:10.1186/s40168-015-0109-2) contains supplementary material, which is available to authorized users.

## Background

In the US, diarrheal diseases are mostly caused by 31 different foodborne pathogens and were reported to contribute to 9.4 million infections, 55,961 hospitalizations, and 1351 deaths each year [1]. Among the bacterial infections reported to FoodNet sites between 2000 and 2008, nontyphoidal *Salmonella* spp. predominated (11 %) followed by *Clostridium perfringens* (10 %) and *Campylobacter* spp. (9 %). Shiga toxin-producing *Escherichia coli* (STEC) O157 and non-O157 also caused 0.7 and 0.6 % of infections, respectively, while *Shigella* spp. caused 1.4 % during this same time frame [1]. In humans, these pathogens can result in asymptomatic colonization, nonbloody diarrhea, or hemorrhagic colitis [2], though they have also been linked to more severe complications. STEC, for example, can cause hemolytic uremic syndrome (HUS), which may result in kidney failure and death, while *C. jejuni* can cause neuropathies (e.g., Guillain Barré Syndrome) and paralysis [3, 4] in a subset of patients. Although the virulence and characteristics of a given pathogen influences disease outcomes, the interaction between the pathogen and host environment is also critical. Little is known, however, about how the microbiota impacts enteric disease susceptibility, severity, and recovery.

Human intestinal communities were previously suggested to comprise >10^12^ microbes/ml [5] with key functions including protection from pathogen proliferation, synthesizing essential nutrients, metabolizing by-products, processing indigestible products from the human diet, and immunomodulation [6, 7]. Members of two phyla, the *Bacteroidetes* and *Firmicutes*, have been found to dominate in the intestines of healthy individuals, though considerable variation has been observed [8]. Prior studies have also demonstrated that intestinal communities are stable over time [9] and that several factors including diet, lifestyle, age, probiotic and antibiotic use, infection, and chronic conditions [10–12], can significantly alter the diversity and abundance of the intestinal microbiota. In patients with inflammatory bowel disease (IBD), for instance, a decrease in the abundance of *Bacteroidetes* and *Lachnospiraceae* has been observed [12], while bile acid profiles associated with animal- versus plant-based diets were associated with growth inhibition of *Bacteroidetes* and *Firmicutes* [13]. Decreased abundance of members belonging to both *Bacteroidetes* and *Firmicutes* was suggested to result in enhanced susceptibility to enteric infections as it shifts the microbial balance away from a healthy state. A prior study in mice demonstrated that intestinal communities with a high abundance of species closely related to pathogens were more likely to be affected by those pathogens [14]. Communities with a high abundance of commensal *E. coli*, for example, were more susceptible to inflammation induced by *Salmonella enterica* infections, whereas diverse intestinal communities were more resistant to infection. Commensal microbes were also suggested to alter pathogen virulence gene expression profiles by modifying environmental conditions [15]. These data underlie the importance of intestinal communities in both promoting and inhibiting pathogen proliferation; however, additional studies are needed to identify which communities are most susceptible to and protective against infection and how they change with infection.

Several studies have also demonstrated that intestinal communities are capable of recovering following perturbations. In mice infected with *Citrobacter rodentium*, a model for human enterohemorrhagic and enteropathogenic *E. coli* infections, alterations in the composition and abundance of the intestinal microbiota could be restored to a pre-infection state upon elimination of the pathogen [16]. Similarly, restoration of intestinal communities disrupted during antibiotic use has also been observed soon after antibiotic cessation [17, 18].

Despite the recent interest and research on the intestinal microbiome, few population-based human studies have been conducted to better understand how the microbiota is impacted by enteric pathogens with different lifestyles. Here, we have compared the composition and abundance of intestinal microbes between patients with acute enteric infections and their uninfected family members by 16S rRNA sequencing. We have also examined how the intestinal microbial communities from a subset of patients change up to 14 weeks post-recovery, and have identified specific microbial populations that are correlated with clinical symptoms.

## Results

### Enteric disease cases identified via active surveillance are representative of the Michigan population

As part of the Enteric Research Investigative Network (ERIN) Cooperative Research Center (CRC) at Michigan State University, we developed an active surveillance system to identify patients with enteric infections at four participating hospitals and the Michigan Department of Health and Human Services (MDHHS). To ensure that the ERIN surveillance network is representative of statewide enteric infection frequencies and that our results are generalizable, we compared the frequency of all ERIN cases (*n* = 615) identified between January 1, 2011 and May 7, 2014 to all cases (*n* = 7435) identified throughout the State of Michigan during the same time period. For each pathogen, the frequency of cases was similar even after stratifying by gender, though a difference was observed after stratifying by age group (data not shown). Because the ERIN network targeted hospitals with the largest pediatric populations, we have captured more pediatric cases, particularly those in the youngest age group.

A total of 310 stool samples were included for 16S rRNA sequencing analysis. These stools originated from 200 patients infected with *Campylobacter* (*n* = 71), *Salmonella* (*n* = 66), *Shigella* (*n* = 34), and STEC (*n* = 28); the pathogen was not known for one patient. A total of 75 stools from otherwise healthy family members were also included for comparison, with 64 (85 %) representing 22 families ranging in size between two and six participants.

### Intestinal microbial communities are distinct between patients with enteric disease and healthy family members

**Fig. 1:**
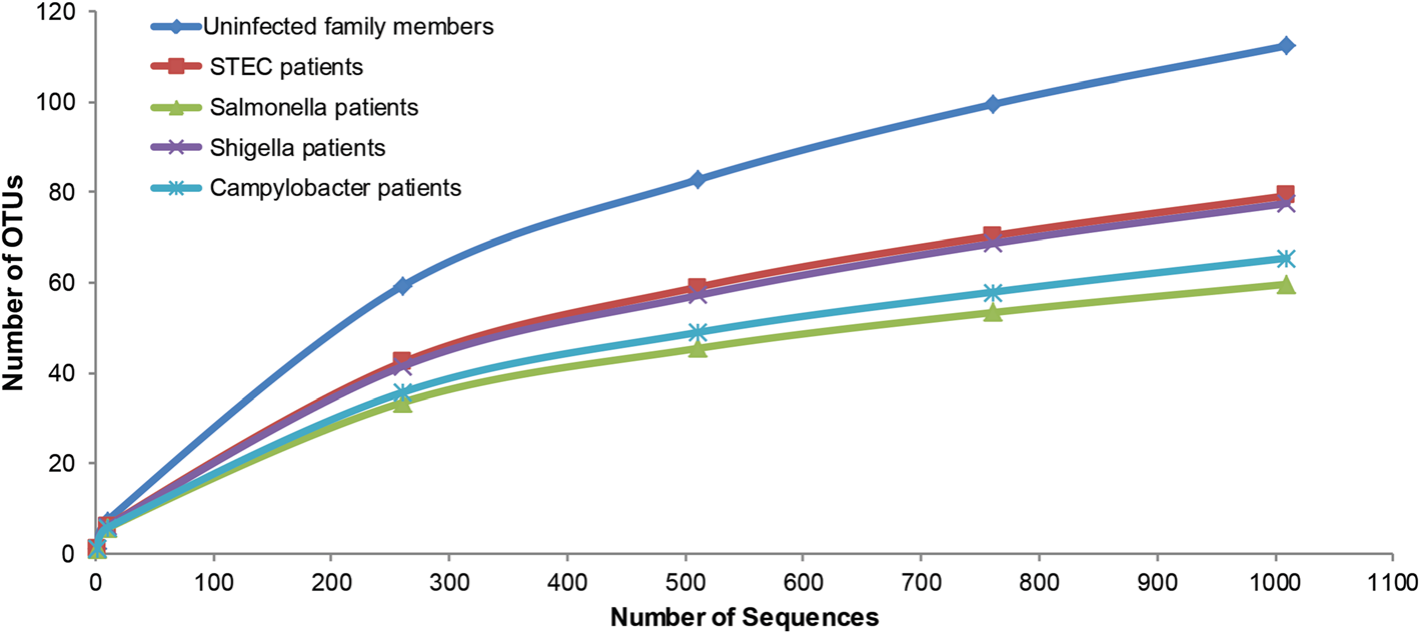
Differences in operational taxonomic units (OTUs) among individuals with and without enteric infections. Rarefaction curve representing the number of OTUs by the number of sequences sampled in intestinal communities from patients with Shiga toxin-producing *Escherichia coli* (STEC), *Salmonella*, *Shigella*, and *Campylobacter* infections relative to uninfected family members

Rarefaction analysis revealed that communities were sampled completely with between 60 to 120 OTUs per sample and at 1000 sequences per sample (Fig. 1). The richness and diversity of the intestinal communities of uninfected individuals (Additional file 1: Table S1) was significantly higher than the patient communities (Additional file 2: Table S2) as estimated by the Chao1 and Shannon indices. For example, Chao1 estimated the microbial communities from all patients to comprise an average of 109 compared to 173 OTUs for uninfected family members. Community composition also differed significantly between the uninfected family members and patients by non-parametric multivariate analysis of variance (NPMANOVA) based on both the Jaccard similarity coefficient (*P* < 0.0001) and Bray-Curtis dissimilarity index (*P* < 0.0001). Moreover, principal coordinates analysis demonstrated tighter clustering of intestinal communities from uninfected family members relative to patient communities based on both the number and abundance of OTUs (Additional file 3: Figure S1). Clustering was not observed among patients infected with different pathogens, though significant differences were observed in the OTU composition and abundance across patients using the NPMANOVA test. *Campylobacter*-infected communities, for example, were significantly different from *Salmonella*, STEC and *Shigella* communities in both composition (Jaccard similarity coefficient *P* = 0.023, *P* = 0.003, and *P* = 0.0004) and abundance (Bray-Curtis dissimilarity index *P* = 0.0003, *P* = 0.01, and *P* = 0.014), respectively. Significant differences were also observed using UniFrac weighted and unweighted distances calculated by the analysis of similarities (ANOSIM) and multivariate ANOVA based on dissimilarities (ADONIS) methods (data not shown).

**Fig. 2:**
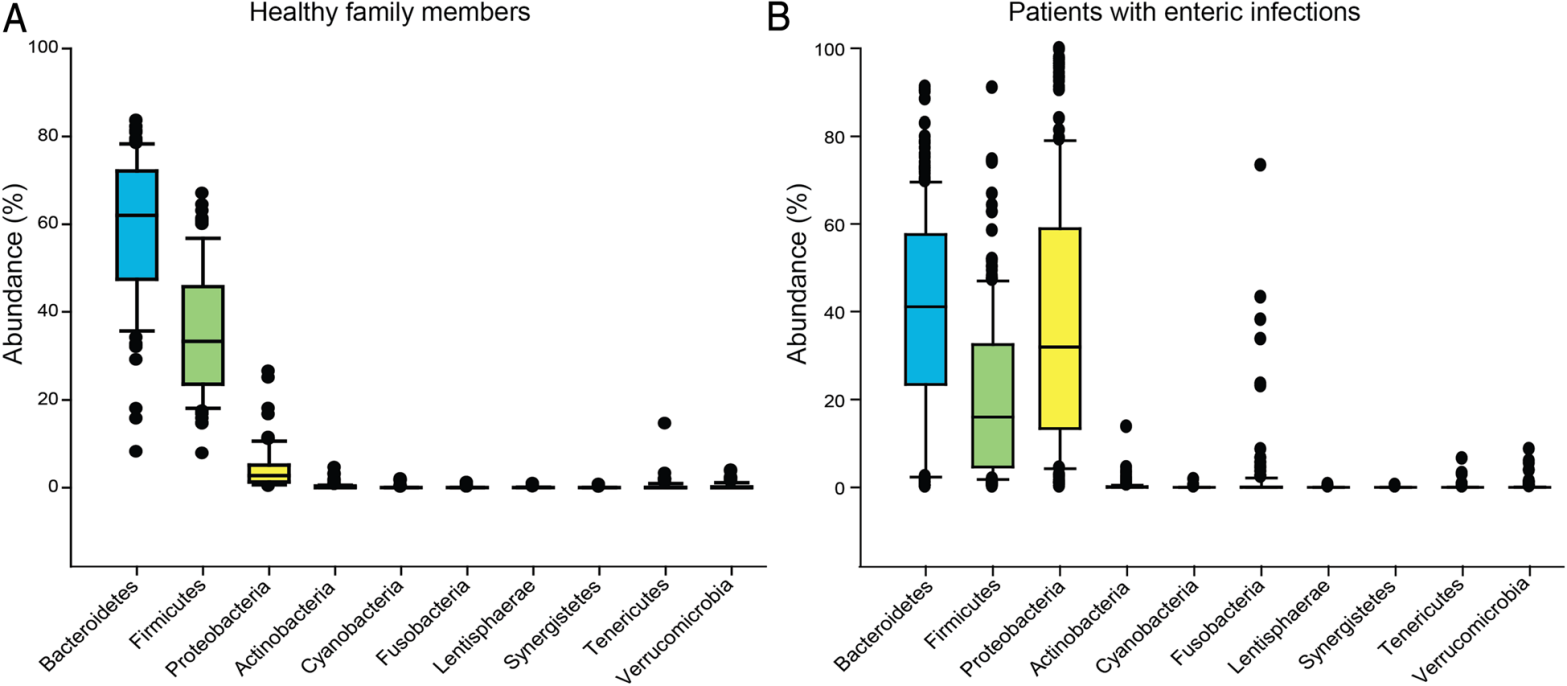
Phyla abundance in communities from **a** healthy family members and **b** patients with enteric infections. Only phyla that were shared between the two groups are highlighted for comparison; *bars* represent averages within each phyla and *black dots* represent outliers

At the phylum level, a higher abundance of *Bacteroidetes* and *Firmicutes* was observed in the uninfected family members compared to the patients, whereas *Proteobacteria* was more abundant in the patient communities (Fig. 2). Indeed, *Proteobacteria* dominated and comprised >20 % of the intestinal community in 121 (61 %) cases; two patients infected with *Salmonella* and STEC had communities comprising 99 % *Proteobacteria*. A total of 28 OTUs representing phylum *Proteobacteria* were classified in the uninfected communities, though variation in OTUs was observed in the communities infected with the four different pathogens (range, 38 to 59 OTUs). Variation in the abundance of *Proteobacteria* was also observed as 37 % of patients with *Campylobacter*, 29 % with *Salmonella*, 18 % with STEC, and 38 % with *Shigella* infections had over 50 % *Proteobacteria*. Among these infected communities, Gammaproteobacteria represented more than 92 % of the *Proteobacteria* detected (Additional file 3: Figure S2), though some variation was observed. Patients infected with *Campylobacter*, for example, had the highest level of Epsilonproteobacteria, which likely represents an overabundance of the pathogen in these cases relative to the others, though Gammaproteobacteria still predominated.

To further demonstrate that the differences observed across patients infected with the different pathogens were not attributable solely to an increase in the abundance of the actual pathogen, we also examined genera abundance and composition across patients. Notably, genus *Escherichia* predominated across communities regardless of the pathogen causing the infection (Additional file 3: Figure S3), though the highest levels were found in STEC cases. The similarity percentages (SIMPER) analysis demonstrated that *Escherichia* was responsible for the highest average dissimilarity (10.9) across the infected communities relative to the uninfected communities. The mean abundance of *Escherichia* in communities infected with *Campylobacter*, *Salmonella*, *Shigella* and STEC was 0.21, 0.14, 0.24, and 0.21, respectively, relative to uninfected communities (0.01). More specifically, the population of Gammaproteobacteria from *Campylobacter* cases represented 29 genera with *Escherichia* comprising 60 % followed by *Trabulsiella* (7 %) and unclassified *Pseudomonadaceae* (7 %). Genus *Campylobacter* was only detected on average in 0.01 % of 41 of the 71 *Campylobacter* cases, which represented 3.7 % of the total *Proteobacteria* levels. *Salmonella* cases had 27 genera represented from Gammaproteobacteria with *Escherichia* and *Trabulsiella* predominating at 45 and 21 %, respectively, followed by *Citrobacter* (12 %) and unclassified *Enterobacteriaceae* (10 %). Only 22 of the 66 *Salmonella* cases had detectable levels (average 0.002 %) of genus *Salmonella*, which represented 0.52 % of the total *Proteobacteria* levels. For STEC cases, 21 genera were identified with *Escherichia* comprising 77 % of the Gammaproteobacteria population identified, which represented the total level of *Proteobacteria* detected in these cases. A total of 23 genera were detected in *Shigella* cases with most belonging to *Escherichia* (69 %), unclassified *Pseudomonadaceae* (8 %), and *Klebsiella* (6 %). Genus *Shigella* was not detected in any of the cases, though unclassified *Enterobacteriaceae* was found at an average of 0.007 %. None of the cases had levels of unclassified *Enterobacteriaceae* exceeding 10 %. Together, these data demonstrate that the distribution of genera differed among the intestinal communities from patients, which was also indicated in the NPMANOVA analysis; however, *Escherichia* predominated in most infected communities regardless of the pathogen causing the infection.

Similar findings were observed utilizing the linear discriminant analysis (LDA) effect size (LEfSe) method [19], which identified 14 microbial features (LDA > 2.4) to be differentially abundant in the patient communities relative to uninfected communities (Additional file 3: Figure S4). Among infected versus uninfected communities, the most abundant features at the family level were *Enterobacteriaceae* followed by *Pateurellaceae*, *Lactobacillales*, *Streptococcaceae*, and *Lachnospiraceae*. At the genus level, *Bacteroides* dominated in 86.7 % of the samples from healthy family members with a range between 6 and 75 %, while *Prevotella* were found in 13.3 % with a range of 16 to 65 %. Among the intestinal communities from healthy individuals, a total of 38 microbial features were differentially abundant. At the family level, most of these features were classified as *Rikenellaceae*, *Bacteroidaceae*, *Ruminococcaceae*, *Porphyromonadaceae*, *Bifidobacteriaceae*, *Alcaligenaceae*, *Odoribacteraceae*, *Barnesiellaceae*, and *Lachnospiraceae*. The genera *Roseburia*, *Lachnospira*, and *Blautia* of the *Lachnospiraceae* family were most abundant in healthy family members compared to patients. Overall, no microbial features were differentially abundant in the communities of patients infected with different pathogens.

### Hierarchical clustering indicates that intestinal microbial communities from patients with enteric infections are similar

**Fig. 3:**
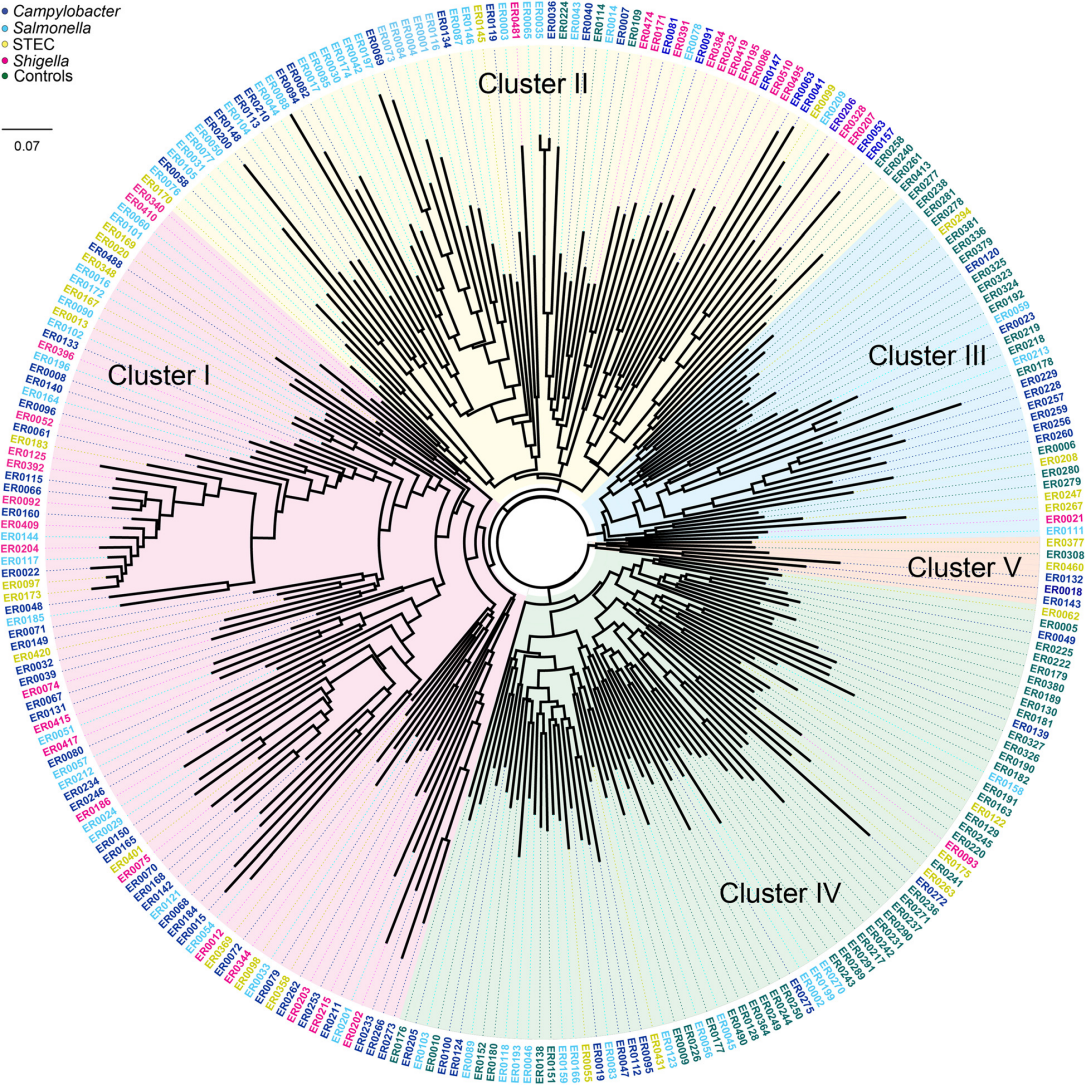
Microbiota profiles among intestinal communities from patients with and without enteric infections. The dendrogram was constructed using the Neighbor-joining method based on the Bray-Curtis dissimilarity index with 1000 bootstrap replications. Hierarchical clustering identified five distinct groups (clusters I–V). Communities from healthy family members (Controls) are labeled in green outside the phylogeny, while patients infected with *Salmonella*, *Campylobacter*, *Shigella*, and Shiga toxin-producing *E. coli* (STEC) are represented by *different colors*

Microbial community composition was examined based on the Bray-Curtis dissimilarity index to compare microbiota profiles between patients (infected communities) and healthy family members (uninfected communities). Five distinct clusters were identified with the infected and uninfected communities separating into different sections of the neighbor-joining tree (Fig. 3). Specifically, 77 % (*n* = 153) of the infected communities belonged to clusters I or II, whereas 91 % (*n* = 68) of the uninfected communities belonged to clusters III and IV. Based on the placement within the dendrogram, communities of cluster V, which originated from one uninfected individual and five patients, were grouped together with communities belonging to clusters III and IV for subsequent analyses. Overall, intestinal communities from patients were significantly more likely to group within clusters I or II, while uninfected communities grouped within clusters III–V (likelihood ratio chi-square (*χ*^2^) = 109.2; *P* = <0.0001).

At the phyla level, clusters I/II had significantly different microbiota profiles in both the weighted and unweighted analysis (NPMANOVA, *P* < 0.0001) when compared to clusters III–V. A significantly higher average percentage of phylum *Proteobacteria* (42 %) was observed in clusters I/II relative to clusters III–V (6 %), while *Bacteroidetes* (38 %) and *Firmicutes* (17 %) were more common in the latter three clusters. Among the uninfected communities belonging to clusters III–V, the abundance of *Bacteroidetes* (59 %) and *Firmicutes* (33 %) was even higher. Significant differences were also observed at the genus level. Intestinal communities representing clusters I and II (*n* = 160) were dominated by *Escherichia*, *Trabulsiella*, *Citrobacter*, unclassified *Pseudomonadaceae*, or *Bacteroides*, whereas communities of clusters III–V (*n* = 115) were dominated by *Bacteroides* or *Prevotella*. Notably, *Escherichia* was significantly more abundant in communities belonging to clusters I and II compared to communities of clusters III, IV, and V as were *Trabulsiella* and unclassified *Enterobacteriaceae* (*P* < 0.0001). *Bacteroides*, *Parabacteroides*, *Faecalibacterium*, *Roseburia*, *Ruminococcus*, *Suturella*, and unclassified *Lachnospiraceae*, however, were significantly more abundant in clusters III–V (Additional file 3: Figure S5). Collectively, these data highlight the differences in the composition and abundance of intestinal microbes across cluster groups and suggest that the microbiota profiles are more similar among patients with enteric infections. No differences in cluster classification were observed across patients infected with different pathogens (Fig. 3).

### Specific microbiota profiles are associated with demographics and clinical symptoms

To determine whether differences in the intestinal microbiota profiles were associated with disease severity and other patient characteristics, we evaluated epidemiological data by the cluster groups defined in the Neighbor joining phylogeny (Fig. 3). No significant associations were identified between the microbiota profiles and age or gender among all individuals examined; however, after omitting uninfected communities from the analysis, male patients were significantly more likely to have profiles representing clusters I/II than clusters III–V (Table 1). Children less than 18 years of age were also more likely to have cluster I/II intestinal microbiota profiles, but the association was not statistically significant. Importantly, several symptoms were more frequently reported by patients with microbial communities belonging to clusters I/II. Among the patients who had reported vomiting or fever, for instance, 85 and 83 %, respectively, had intestinal communities belonging to clusters I/II. A similar association was observed for bloody diarrhea, which was reported by patients during the time of their infection. Among 179 patients with data available, individuals reporting bloody diarrhea were twice as likely to have cluster I/II intestinal communities compared to individuals reporting nonbloody diarrhea (*P* = 0.04). Indeed, only 14 of the 85 patients (17 %) with bloody diarrhea had intestinal communities belonging to clusters III–V. No association was observed between specific intestinal microbiota profiles and length of hospitalization, patient age group, or history of nausea, headache, fatigue, and chills.

**Table 1:** Univariate analaysis highlighting the association between intestinal microbiota profiles and host factors among 200 patients with enteric infections

	Community clusters I and II (*n* = 152)		Community clusters III–V (*n* = 47)		
Characteristics	No. (%) of individuals		No. (%) of individuals		*P* value^a^
Age group					0.17
0–2 years (*n* = 31)	25	(16.5)	6	(12.8)	
3–18 years (*n* = 55)	44	(29.0)	11	(23.4)	
19–52 years (*n* = 76)	58	(38.2)	18	(38.3)	
>52 years (*n* = 37)	25	(16.5)	12	(25.5)	
Gender					0.02
Male (*n* = 104)	86	(57.3)	18	(38.3)	
Female (*n* = 93)	64	(42.7)	29	(61.7)	
Symptoms					
No diarrhea (*n* = 3)	2	(1.5)	1	(2.3)	0.03
Diarrhea only (*n* = 91)	63	(46.3)	28	(65.1)	
Bloody diarrhea (*n* = 85)	71	(52.2)	14	(32.6)	
ᅟ					
No vomiting (*n* = 118)	84	(62.7)	34	(79.1)	0.05
Vomiting (*n* = 59)	50	(37.3)	9	(20.9)	
ᅟ					
No fever (*n* = 62)	42	(33.9)	20	(54.1)	0.03
Fever (*n* = 99)	82	(66.1)	17	(46.0)	
Length of hospitalization					0.67
None (*n* = 126)	94	(66.2)	32	(74.4)	
1–2 days (*n* = 19)	18	(12.7)	1	(2.3)	
≥3 days (*n* = 40)	30	(21.1)	10	(23.3)	
Type of pathogen (*n* = 199)					0.05
*Campylobacter* (n = 71)	55	(36.2)	16	(34.0)	
*Salmonella* (*n* = 66)	49	(32.2)	17	(36.2)	
Shiga toxin *E. coli* (*n* = 28)	16	(10.5)	12	(25.5)	
*Shigella* (*n* = 34)	32	(21.1)	2	(4.3)	

Percentages represent the frequency of each characteristic out of the total number of patients per cluster group. Denominators for host characteristics do not always add up to the total number of infected individuals (*n* = 200) as some data were missing. Symptom data was missing for up to 23 patients. Because most patients with missing data had microbiota communities belonging to clusters I/II, it is not likely that inclusion of the missing data would impact the direction of each significant association

^a^The likelihood ratio chi-square or Mantel-Haenszel chi-square (for variables with more than two outcomes) were used to highlight differences between clusters I–II and clusters III–V

While adjusting for age, gender, and intestinal microbiota profile, logistic regression analyses demonstrated that patients with intestinal communities belonging to clusters I/II were significantly more likely to have bloody diarrhea (odds ratio (OR), 2.3; 95 % CI, 1.12, 4.82). Male gender, however, resulted in a protective effect (OR, 0.5; 95 % CI, 0.28, 0.91), and no association was observed for length of hospitalization. Addition of pathogen type to the logistic regression model resulted in similar associations for gender and cluster, but also showed that patients with STEC infections were significantly more likely to have bloody diarrhea when compared to patients with *Salmonella*, *Shigella*, and *Campylobacter* (OR, 3.7; 95 % CI, 1.37, 9.79). Nonetheless, these data suggest that individuals with more severe disease, as measured by the presence of bloody diarrhea, were more likely to have intestinal communities with similar profiles regardless of age and infection by three of the four pathogens.

### The intestinal microbial communities of patients up to 14 weeks post-infection mimics the intestinal communities of healthy family members

**Fig. 4:**
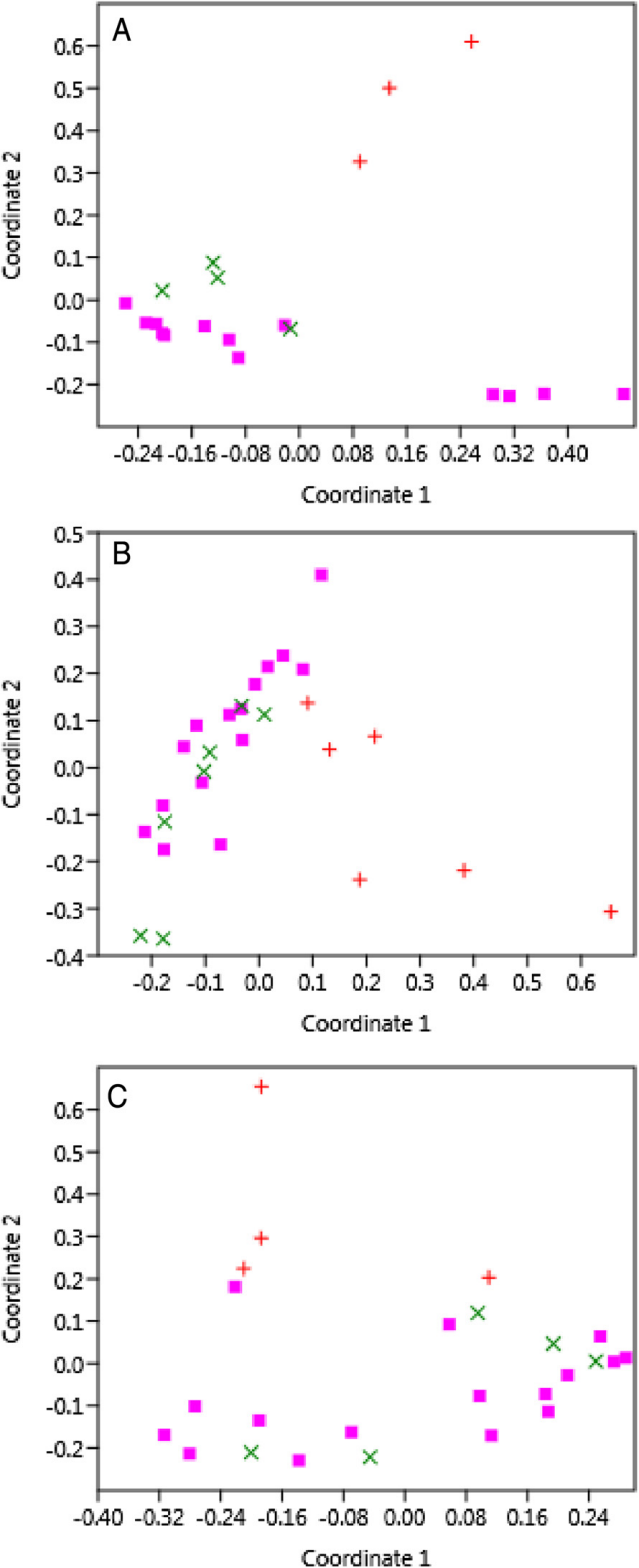
Principle coordinate analysis depicting the shift in microbial communities following recovery. Intestinal communities of 13 patients with **a** *Campylobacter* (*n* = 3), **b** *Salmonella* (*n* = 6), and **c** *Shigella* (*n* = 4) infections were compared during the acute enteric infection (*red*) and post-recovery (*green*). A comparison was also made to the intestinal communities among healthy family members (*pink*). The number of individuals sampled during the acute infection stage did not always equal the number of samples received following recovery due to missing samples or loss to follow-up; hence, the time points were pooled for the analysis

Stool samples from a subset of 13 patients at one to 14 weeks following recovery from *Campylobacter* (*n* = 3), *Salmonella* (*n* = 6), and *Shigella* (*n* = 4) infections were examined to assess how intestinal communities changed following an enteric infection. In each case, a shift in the intestinal community was observed following recovery. Collectively, the recovered intestinal communities had greater richness, evenness, and diversity relative to the communities that were present during the acute infection (Additional file 3: Figure S6). Comparisons were also made between infected intestinal communities, recovered communities, and communities from healthy family members. Principle coordinate analysis based on the Bray-Curtis dissimilarity index demonstrated that recovered communities become more similar to the healthy communities regardless of the pathogen that originally caused the enteric infection (Fig. 4). Notably, the phyla associated with the shift in community composition post-recovery were *Bacteroidetes*, *Firmicutes*, and *Proteobacteria*. At the time of the acute infections, *Bacteroidetes* and *Firmicutes* were significantly less abundant than in both the communities from recovered patients and healthy family members (*P* < 0.0001), while the level of *Proteobacteria* was significantly greater (*P* < 0.0001) (Additional file 3: Figure S7). No significant difference was observed in the abundance of *Bacteroidetes* and *Firmicutes* in the recovered communities relative to communities from healthy family members, though *Proteobacteria* abundance was still significantly greater in recovered communities post-infection (*P* = 0.025). Such differences are likely attributable to variation in the time that each recovered patient was evaluated as patients submitted a follow-up sample between 1 and 14 weeks post-infection. Indeed, *Proteobacteria* levels decreased over time across the 14 week follow-up period in all patients regardless of the pathogen causing each infection. At the genus level, this decrease in *Proteobacteria* was mostly attributable to a decrease in *Escherichia*, which coincided with an increase in *Bacteroides*, *Prevotella*, *Parabacteroides*, and *Rikenellaceae* in phylum *Bacteroidetes* and *Faecalibacterium* belonging to phylum *Firmicutes* (Additional file 3: Figure S8).

## Discussion

Although the microbial communities of the human gut have been found to be both complex and diverse with considerable variation across individuals [6, 8], our examination of the intestinal microbiota from patients with enteric infections has shown a decreased diversity relative to intestinal communities from healthy individuals. This finding is not surprising in that individuals suffering from acute diarrhea can lose up to 200 g of stool and fluids each day [20], thereby inducing a significant change to the intestinal microbiome. Through this study, however, we have demonstrated that patients with acute enteric infections caused by four different bacterial pathogens have similar deficiencies in the abundance of certain intestinal microbes. It is therefore likely that specific microbial populations are more commonly selected for during the course of an infection, and that restoring intestinal health relies on the time it takes these intestinal communities to recover and shift back to a pre-infection or healthy state.

Irrespective of the bacterial pathogen that caused each infection, a greater abundance of *Proteobacteria* and lower abundance of *Bacteroidetes* and *Firmicutes* was detected in the patients’ (infected) intestinal communities when compared to the uninfected communities of healthy family members. Both *Bacteroidetes* and *Firmicutes* have previously been associated with intestinal health [8], and all patients in this study had significantly lower levels of both phyla relative to uninfected family members. In addition, among the 200 patients with enteric infections, 63.5 % of the population had communities comprising >20 % *Proteobacteria* compared to an average of only 4.3 % of the healthy individuals (*n* = 75). Although the total number and relative abundance of OTUs uncovered in the intestinal communities from individuals were lower than in prior studies, all stool samples were preserved in the same conditions with Cary-Blair media, which had little effect on the presence and abundance of specific community members following experimental inoculation of cattle feces (Additional file 3: Figure S9). Because each intestinal community was similarly affected by the preservation media, differences could be identified and comparisons could be made across individuals with and without disease. Likewise, given the challenges associated with DNA recovery from gram-positive bacteria, it is possible that the abundance of specific community members may have been underestimated. Since the same DNA isolation protocol, which incorporated a recommended mechanical disruption step [21], was utilized for each stool sample and high frequencies of gram-positive bacteria were recovered from many samples, we do not expect that the differential recovery of these bacteria in the uninfected versus infected samples contributed to bias in this study. In addition, our results showing increased levels of *Proteobacteria* in infected communities are similar to those generated in prior studies and suggest that enhanced *Proteobacteria* may be indicative of a diseased state that commonly occurs during enteric infections or following perturbations in the microbiota. One previous study, for example, observed an overgrowth of *Enterobacteriaceae* after *C. rodentium* infection in mice due to microbiota depletion and induction of host inflammatory responses [16], while patients with inflammatory bowel disease had increased levels of *Enterobacteriaceae* [22] or *Proteobacteria* [23] relative to healthy controls.

Since the majority of *Proteobacteria* represented genus *Escherichia* among all patients with enteric infections regardless of the bacterial pathogen causing the infection, it is likely that this microbial population is more frequently selected for during an acute enteric infection. Factors that contribute to an increase in specific microbes over others, however, are not fully understood, though we do know that microbiota depletion and host immune responses are likely to be critical. *Salmonella typhimurium*, for instance, was shown to outcompete the host microbiota by exploiting host inflammatory immune responses [24] and utilizing luminal thiosulfate from the reactive oxygen species generated during inflammation [25]. Similarly, enterohemorrhagic *E. coli* and enteropathogenic *E. coli* were shown to accept nitrate produced by host innate immune cells for use as energy via anaerobic respiration to enhance survival in the inflamed mouse intestine [26]. Because members of *Bacteroidetes* and *Firmicutes* cannot utilize nitrate, it was suggested that the microbial balance becomes disrupted by promoting overgrowth of both pathogenic and commensal *E. coli* following inflammation [26]. Our study provides additional support for these findings and suggests that infection with not only pathogenic *E. coli* induces an increase in *Escherichia* levels within infected intestinal communities. Increases were also observed in patients infected with *Campylobacter*, *Salmonella* and *Shigella*. Interestingly, STEC patients had the highest levels of *Escherichia* as compared to patients with *Campylobacter*, *Shigella*, and *Salmonella* infections, suggesting that both the pathogenic STEC and commensal *Escherichia* population were selected for at the time of the infection. In addition, the decrease in *Proteobacteria* levels among the 13 patients following recovery from infection demonstrates that the increase in *Proteobacteria* levels occurred during the acute infection and that healthy levels are significantly lower. A study of Cholera reported similar findings as *Proteobacteria* was more abundant during the acute stage of Cholera infections in nine children when compared to 28 days post-infection [27].

In addition to the decreasing level of *Proteobacteria* belonging to genus *Escherichia* identified in a subset of patients post-infection, we also observed significant increases in phyla *Firmicutes* and *Bacteroidetes*, which were not significantly different from levels in healthy family members. These results are consistent with prior reports demonstrating that intestinal communities recover following perturbations. Several studies, for instance, have documented a shift in intestinal communities during periods of antibiotic use with recovery of those communities occurring soon after antibiotic cessation [17, 18]. Other studies have also shown that disruptions in intestinal microbial communities can result in enhanced susceptibility to pathogen colonization, invasion, and infection [15]. Future studies, however, are needed to determine whether certain communities are more susceptible to severe perturbation than others. Here, we observed greater increases in *Proteobacteria* and *Escherichia* levels in some patients and not others, though logistic regression analyses did not link either high *Proteobacteria* or *Escherichia* abundance with clinical symptoms. Conversely, we found that patients with intestinal community profiles belonging to clusters I/II were significantly more likely to report a history of bloody diarrhea than patients with distinct communities. These patients were also significantly more likely to be male, suggesting that the complete microbiota profile of the cluster I/II communities are more important for dictating disease outcomes during an acute infection than the increased level of *Escherichia*. Because our sequencing analyses focused on 16S rRNA genes, a more comprehensive metagenomic analysis is warranted between these patient and family member communities to better define which microbes comprise clusters I/II and III–V, and identify relationships between complete microbiome profiles and disease severity.

Collectively, the data presented herein demonstrate that intestinal microbial communities become affected in a similar way among patients with enteric infections regardless of the pathogen type and age of the patient. Perturbations of specific communities (e.g., Cluster I/II) that are characterized by an increase in *Escherichia* are more likely to have more severe symptoms; hence, it is possible that these communities with a higher abundance of *Escherichia* take longer to recover to a healthy state. Future studies should therefore focus on characterizing the population of *Escherichia* as it is not clear whether there is overgrowth of one strain type or if the population is diverse consisting of genetically distinct strains. Based on our data, it is also important to consider future treatment strategies that involve the use of probiotics or therapeutics that aim to increase the abundance of beneficial microbes comprising *Bacteroidetes* and *Firmicutes* and more rapidly decrease *Proteobacteria* populations. Such strategies could potentially decrease the burden of enteric infections and facilitate a quicker recovery.

## Conclusions

Enhancing our understanding of how intestinal microbial communities change during an enteric infection and identifying factors that influence these changes are important for the development of novel prevention and treatment strategies. In our analysis of 200 intestinal communities from patients and 75 healthy family members, we found that specific intestinal microbiota are more common among patients during an acute enteric infection regardless of the type of enteric pathogen. Specifically, the intestinal microbial communities from patients were more frequently dominated by *Proteobacteria* and not *Bacteroidetes* and *Firmicutes*, which represent dominant commensals and the most common phyla in human intestinal communities. Patients with similar microbiota profiles were more likely to have severe disease outcomes, suggesting that increases in specific microbe populations, which were primarily attributable to *Escherichia*, occur during an infection. This finding is supported by the result that patient intestinal communities recovered over time to mimic those communities of healthy family members, which were classified by an increase in phyla *Bacteroidetes* and *Firmicutes* and decrease in *Proteobacteria* belonging to genus *Escherichia*. Future enteric disease prevention strategies could therefore aim to decrease intestinal *Escherichia* levels at the time of an acute infection while simultaneously boosting microbes including *Bacteroidetes* that are most important for restoring intestinal health.

## Methods

### Study design

An active surveillance system was created through the MDHHS Bureau of Laboratories to identify 200 patients infected with *Campylobacter*, *Salmonella*, *Shigella*, and STEC at four participating Michigan hospitals. Surveillance began on January 1, 2011. Stools from each patient were added to Cary-Blair transport media and submitted to the MDHHS for de-identification, culture, and transport to MSU for community DNA isolation and 16S rRNA sequencing. Family members of patients (*n* = 75) were enrolled by phone and asked to submit a stool specimen preserved in Cary-Blair transport media and to complete a questionnaire containing demographic information, exposures (e.g., animal contacts, travel history), and signs of illness including duration, type, and severity of clinical symptoms. Such information was also available for each patient through the Michigan Disease Surveillance Network (MDSS) maintained by the MDHHS Bureau of Epidemiology. A subset of 13 patients was also re-contacted post-infection for stool submission and questionnaire completion. Each participant was required to provide informed consent prior to enrollment and was given a monetary incentive after samples were received at MDHHS. All protocols were approved by the Institutional Review Boards at MSU (IRB# 10-736SM), the MDHHS (842-PHALAB), and each of the four participating hospitals (RR#12170, University of Michigan; 202749-6 Sparrow Health System; 2011-039 Spectrum Health, and 022111MP4E, Wayne State University/Detroit Medical Center).

### DNA isolation and sample preparation

Stool samples were processed soon after receipt by both MDHHS and MSU and time period from collection to processing was noted. Following homogenization and centrifugation, aliquots of stool were stored in triplicate at −80 °C until further use. A total of 310 stools were examined in this analysis, which represent 41.8 % of the total number of stools (*n* = 741) received via the ERIN surveillance system through May 7, 2011. Stool DNA recovery (260/280 nm) and yield (ng/μl) were initially compared using the QIAamp DNA Stool Mini Kit (QIAGEN; Valencia, CA) and the MoBio Fecal Isolation Kit (MoBio; Carlsbad, CA) and standardized using the NanoDrop (Thermo Fischer Scientific). Four stools were compared using the manufacturer’s instructions, except for the addition of a denaturation step (95 °C) followed by bead beating for the QIAamp kit to enhance DNA isolation of gram-positive bacteria as described [21]. Samples were also evaluated for changes before and after homogenization. Overall, the QIAamp kit resulted in better quality yields, enhanced diversity (Additional file 3: Figure S10), and better recovery of phyla representing gram-positive bacteria (Additional file 3: Figure S11). Hence, the QIAmp protocol was used for the remaining 306 samples. No differences were observed between communities from the same individual before and after homogenization.

Because all stool samples were transported in Cary-Blair media to preserve the pathogens for culture, we wanted to ensure that the preservation media did not alter the composition of the microbiota (Additional file 3: Figure S9). To do this, we incubated cattle feces obtained through a different study with and without Cary-Blair at 4 °C for up to 4 days; we did not have access to unpreserved human samples. Four days represented the average length of time for human samples to reach our laboratory from the MDHHS prior to isolating community DNA. Pyrosequencing was performed on both sets of samples for comparison.

### Pyrosequencing and community analysis

Isolated community DNA was amplified for the 16S rRNA gene spanning the V5–V3 regions using universal primers 357F and 926R tagged with the 454 universal primers A and B at either end with barcodes for multiplexing samples. PCR amplification was performed using AccuPrime^™^ Taq DNA polymerase (Invitrogen^™^) in triplicate with initial denaturation at 95 °C for 2 min followed by 30 cycles of 95 °C for 20 s, 50 °C for 30 s, and 72 °C for 5 min. An amplicon library of 48 communities were prepared for each sequencing run on 454 GS Junior Titanium chemistry, by individually purifying the product using the Agencourt AMPure XP (Beckman Coulter, Inc.) beads, quanitification by pico green assays (Quant-iT^™^ PicoGreen® dsDNA quanititation kit, Invitrogen^™^) followed by normalization and pooling.

### Sequencing analysis

Over 1 million reads were processed in the Quantitative Insights Into Microbial Ecology (QIIME) software [28] and aligned and classified using the Greengenes 16S rRNA gene reference database. Sequence data were subjected to quality trimming, filtering, and noise reduction by QIIME denoiser using the default parameters [28]. The unaligned sequences were eliminated followed by chimeric sequences and singleton removal using the USEARCH quality filtering included in QIIME and the data have been analyzed for microbial community composition, diversity, and richness [29]. OTUs were clustered at 97 % similarity cutoff using USEARCH where 16S rDNA sequences were classified using the Greengenes database. Rarefaction curves were constructed using QIIME [28] based on the observed OTUs in infected and uninfected stool samples, Shannon diversity index, and the Chao1 richness estimator. The OTU biome table from QIIME was used in Mothur to calculate richness and evenness indices with 95 % confidence intervals (Additional file 1: Tables S1 and Additional file 2: Table S2) as described previously [30]. LEfSe was then used to identify differentially abundant microbial features as described previously [19]. UniFrac distances were calculated in QIIME for both abundance and composition and 3D principal coordinate analysis plots were generated using KING (Kinemage, Next Generation) [31].

### Hierarchical clustering analysis and epidemiological analyses

The phylogenetic data from QIIME was further analyzed using Paleontological Statistics Software Package For Education and Data Analysis (PAST) based on the NPMANOVA and to calculate genera important for dissimilarities between groups using SIMPER. Additional 2D principal coordinate analysis plots based on both the Bray-Curtis and Jaccard similarity coefficients were also constructed for a weighted and unweighted analysis (28). Box plots were constructed using SigmaPlot 10.0.

Links to epidemiological data were made after clustering relationships were identified between the stool communities based on abundance profile calculated by Bray-Curtis dissimilarity index in PAST for generation of neighbor-joining tree followed by tree construction in FigTree (http://tree.bio.ed.ac.uk/software/figtree/). We tested for differences in the frequency of clinical characteristics including HUS, diarrhea, bloody diarrhea as well as for various factors including age and gender, across the different microbial community clusters using the likelihood chi-square test or Mantel-Haenszel chi-square (for variables with more than two outcomes) distributions using odds ratios with 95 % confidence intervals performed with Statistical Analysis Software (SAS) (version 9.3).

## Availability of supporting data

Raw sequence data is available at (http://dx.doi.org/10.6084/m9.figshare.1447256), while the metadata and taxonomic classifications have been included as Additional files 4 and 5.

## Additional files

Additional file 1:
**The richness and diversity estimates for intestinal communities from otherwise healthy individuals without enteric infections.** (XLSX 22 kb) Additional file 2:
**The richness and diversity estimates for intestinal communities from patients with enteric infections.** (XLSX 46 kb) Additional file 3:
**Supplemental figures S1-S11.** (DOC 6266 kb) Additional file 4:
**Metadata associated with the individuals included in the study.** (XLSX 16 kb) Additional file 5:
**Operational taxonomic units in the intestinal communities from patients infected with different enteric pathogens and otherwise healthy individuals without enteric infections.** (XLSX 320 kb)
