# Supplementary material for: Intestinal microbial communities associated with acute enteric infections and disease recovery
Source: Microbiome. 2015 Sep 22;3:45. doi: 10.1186/s40168-015-0109-2 (PMC4579588; doi:10.1186/s40168-015-0109-2)
Supplement: Additional file 3: — Supplemental figures S1-S11. (DOC 6266 kb) [file 40168_2015_109_MOESM3_ESM.doc]

**Figure S1**. Principle coordinate analysis based on the **A**) Unweighted UniFrac and **B**) Weighted UniFrac illustrating clustering of the healthy intestinal communities and not the patient communities based on composition and abundance of community members, respectively. Patients infected with different pathogens are represented by different colors.


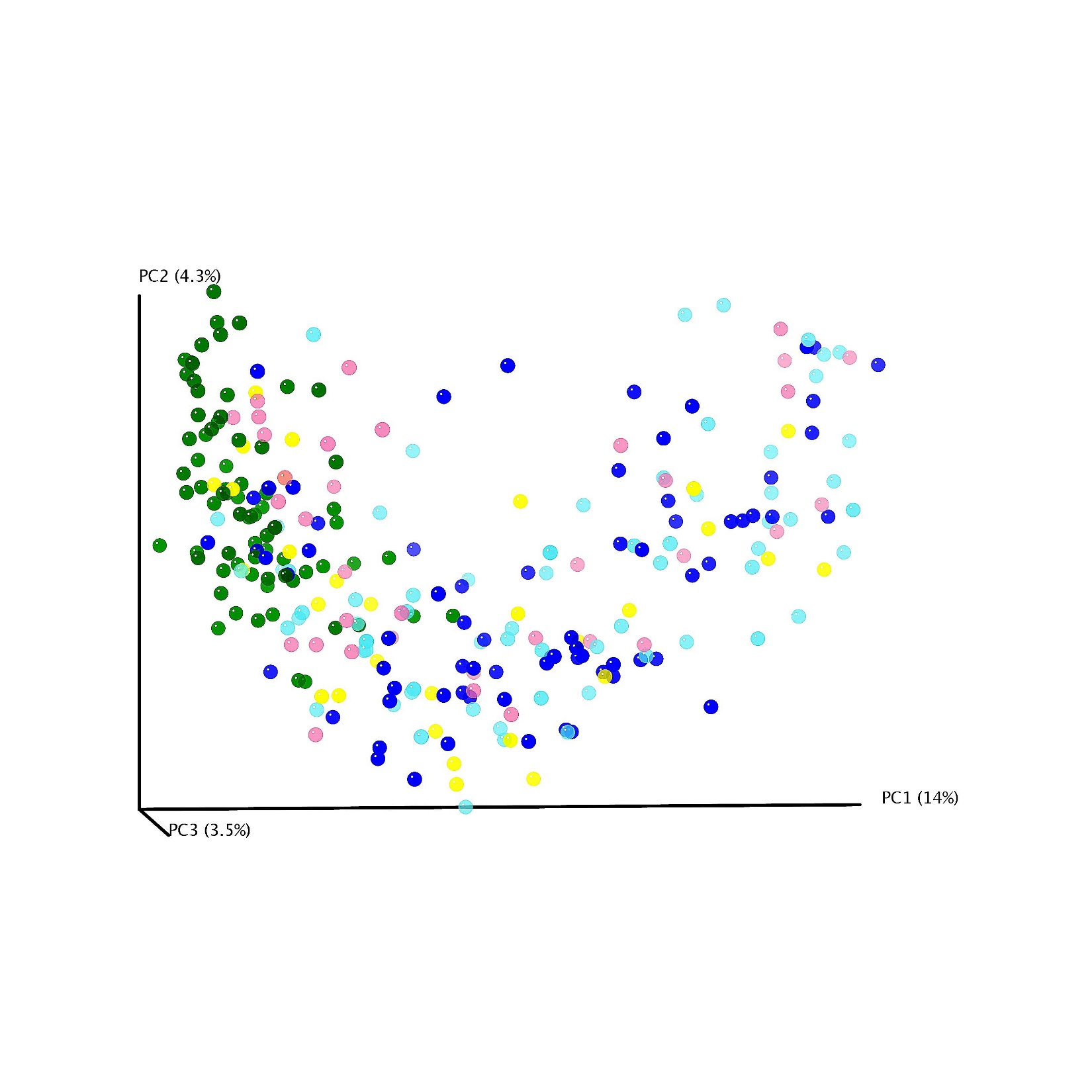

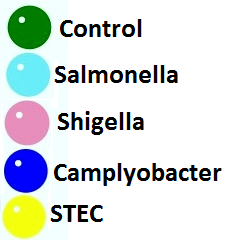
**A.**

**
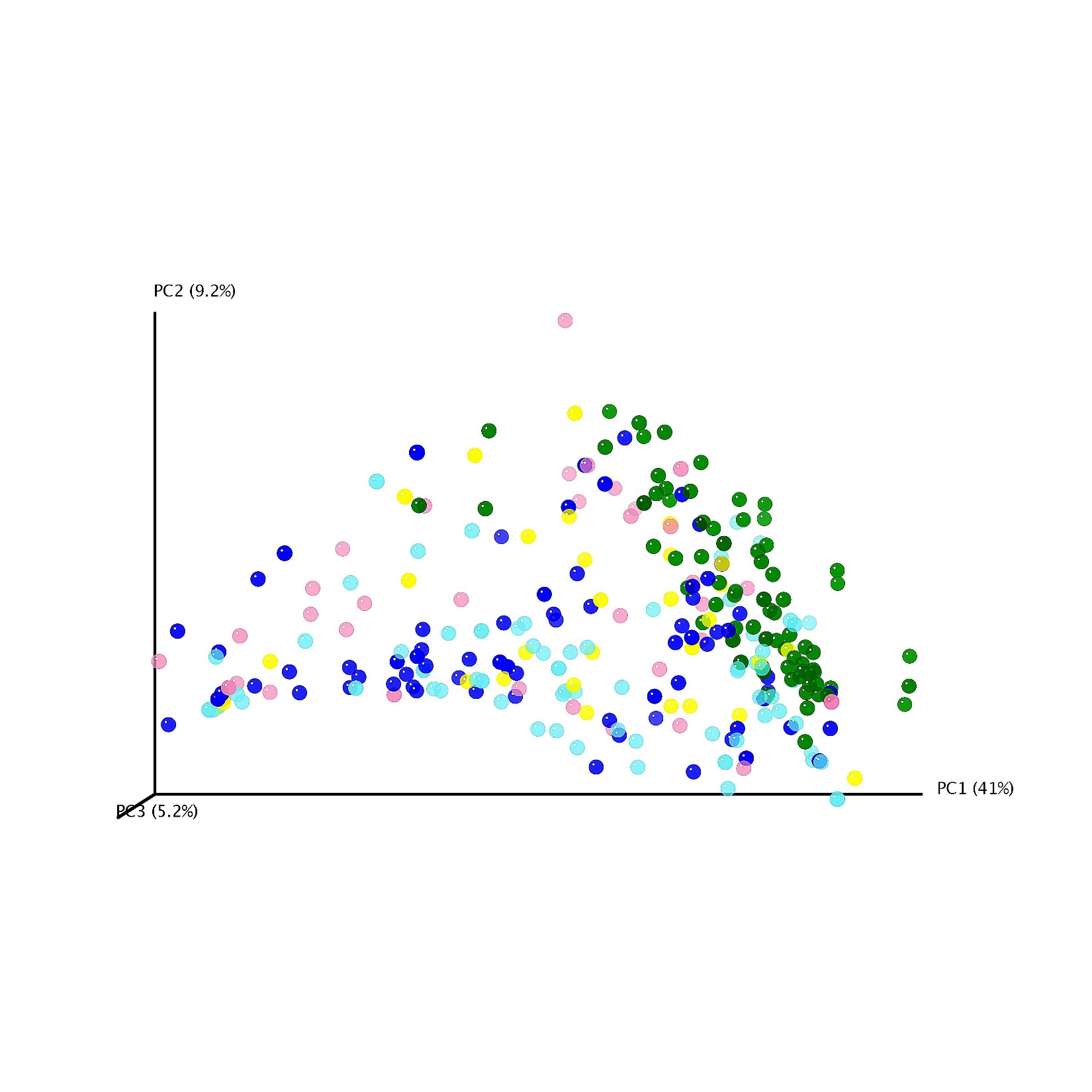
**


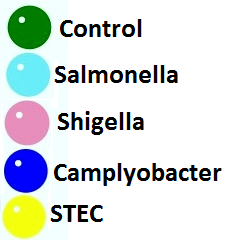
**B.**

**Figure S2**. Distribution of *Proteobacteria* lineages in patients with enteric infections caused by **A**) *Campylobacter*; **B**) *Salmonella*; **C**) *Shigella*; and **D**) Shiga toxin-producing *Escherichia coli* (STEC). Gammaproteobacteria predominated in the intestinal communities of cases irrespective of the pathogen.


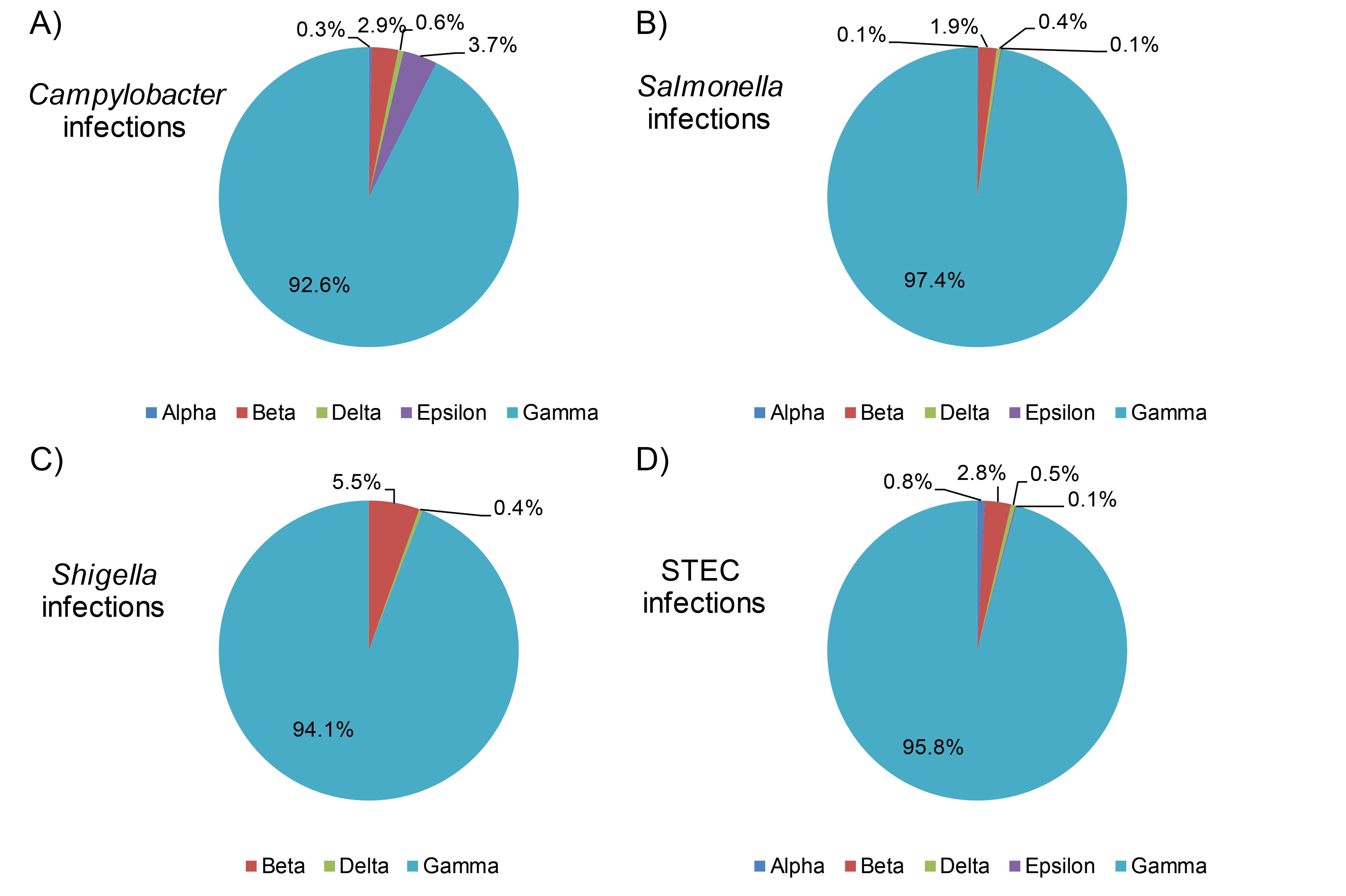


**Figure S3.** Abundance of genera in patients with enteric infections and otherwise healthy family members. *Escherichia* (peach) is more abundant in most patient communities, while *Bacteroides* (purple) predominates in most healthy communities. The distribution of other genera varies considerably across intestinal communities.


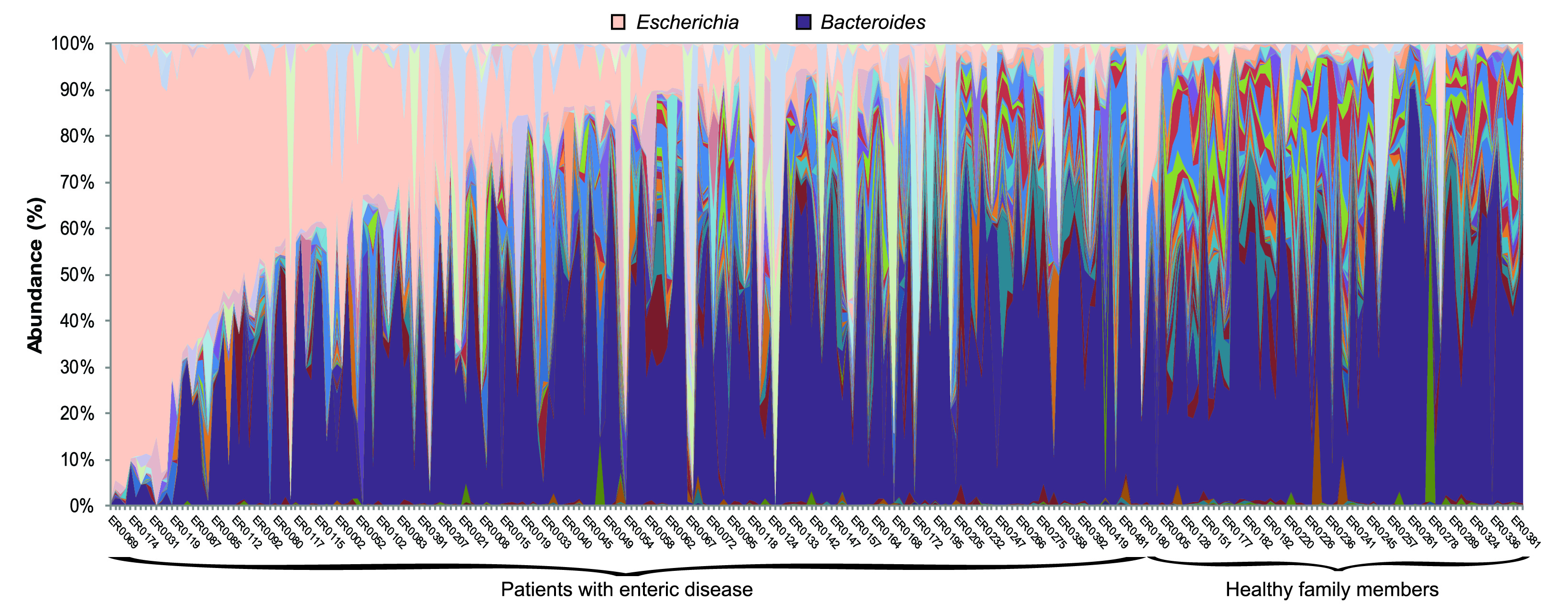


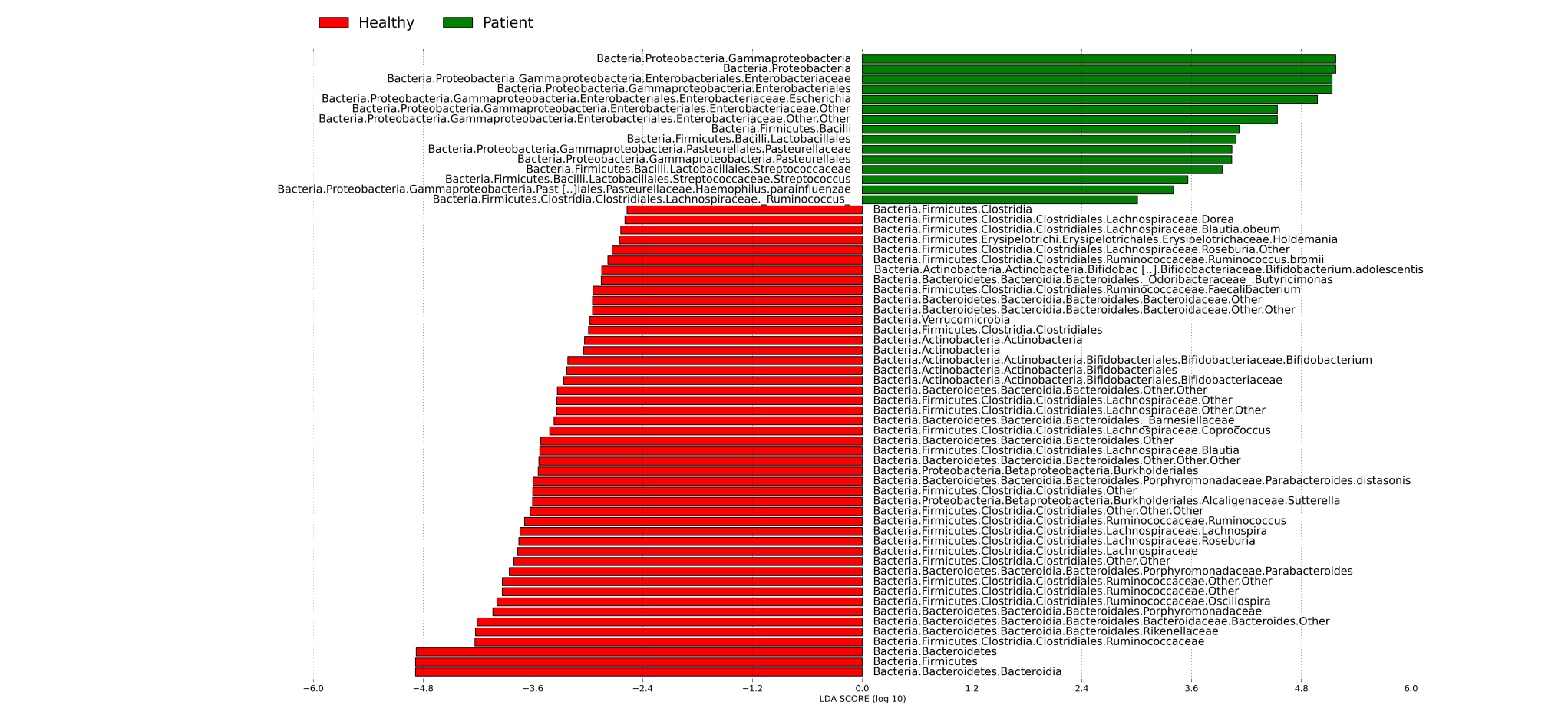
**Figure S4**. Differentially abundant microbial features in the intestinal microbial communities of patients versus healthy family members as determined by linear discriminant analysis (LDA) effect size (LEfSe). A total of 38 microbial features were differentially abundant in uninfected family members as were 14 in the patients (LDA score >2.4).


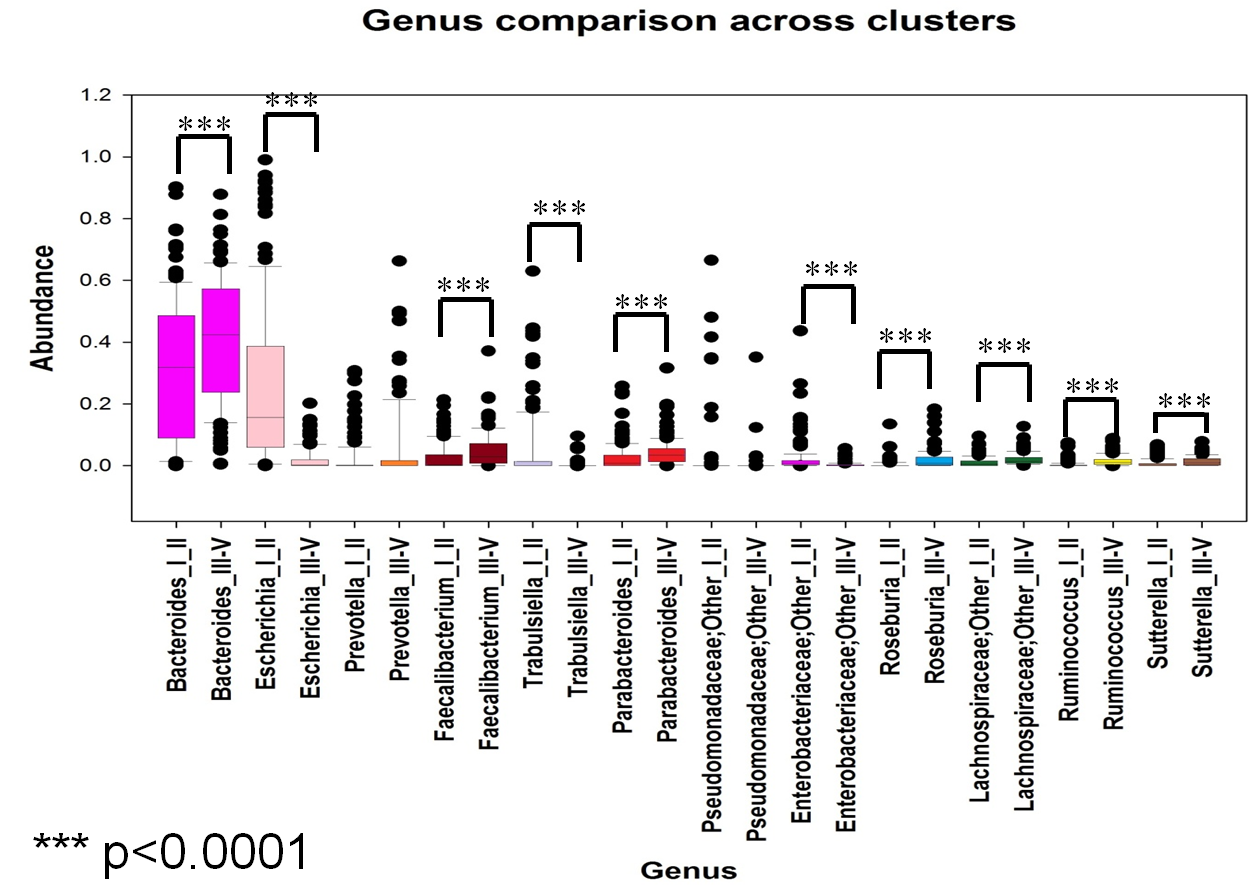
**Figure S5**. Differences in the abundance of dominant genera across the two major clusters (I-II versus III-V) defined by heirarchical clustering.

**Figure S6.** Rarefaction curve highlighting the Shannon diversity by the number of sequences per sample. Communities from samples of patients post infection (blue) had higher diversity as compared to their communities during and active enteric infection (red).


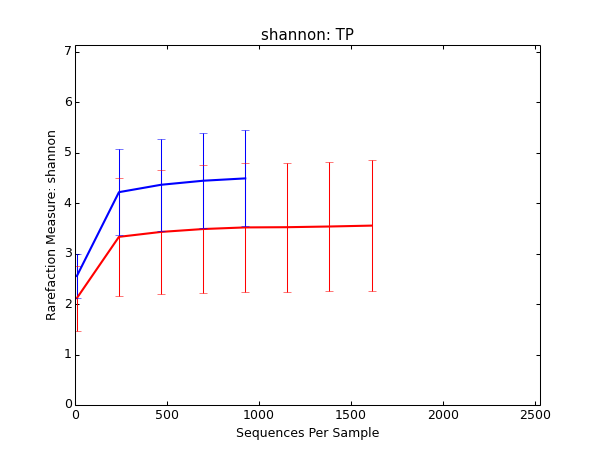

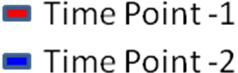


**Figure S7.** Comparison of phyla abundance in 13 patients between time point 1 (TP1) during an active enteric infection and time point 2 (TP2) following recovery of the infection. Abundance of each phylum is also noted for healthy family members (CON) for comparison. Follow up samples representing decrease in *Proteobacteria* population whereas an increase in dominant phyla *Bacteroidetes* and *Firmicutes* is observed.


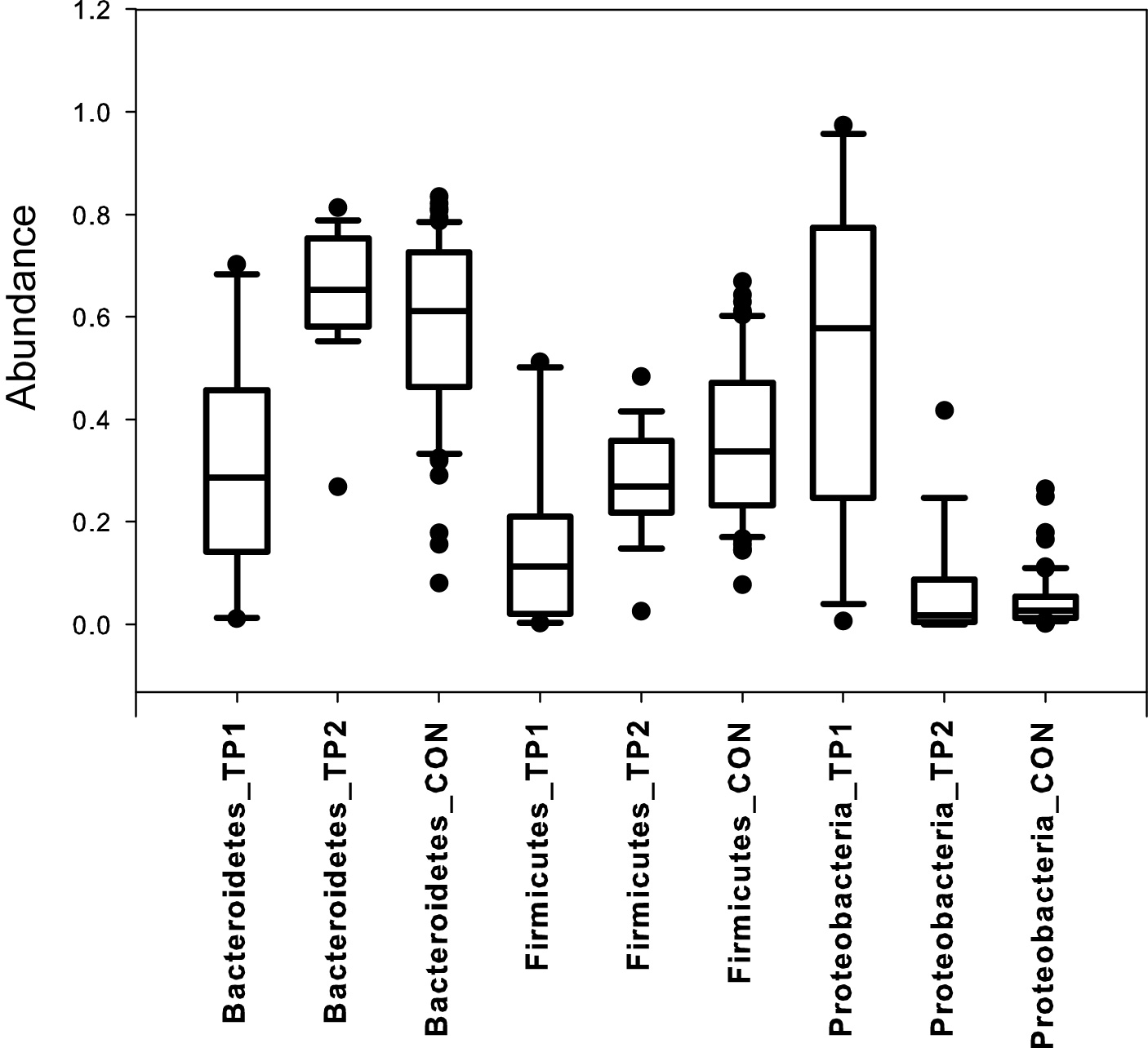


**Figure S8**. Comparison of genera abundance between time point 1 (TP1) during an active enteric infection and time point 2 (TP2) following recovery of the infection. Abundance of each phylum is also noted for healthy family members (CON) for comparison. Any genus contributing to over 2% of dissimilarity across the infected and uninfected samples by SIMPER were included in the plot.

**Figure S9**. Rarefaction analysis representing the number of operational taxonomic units (OTUs) by the number of sequences sampled for 10 bovine-derived fecal samples preserved in Cary-Blair media relative to the same fecal samples without Cary-Blair preservation media.


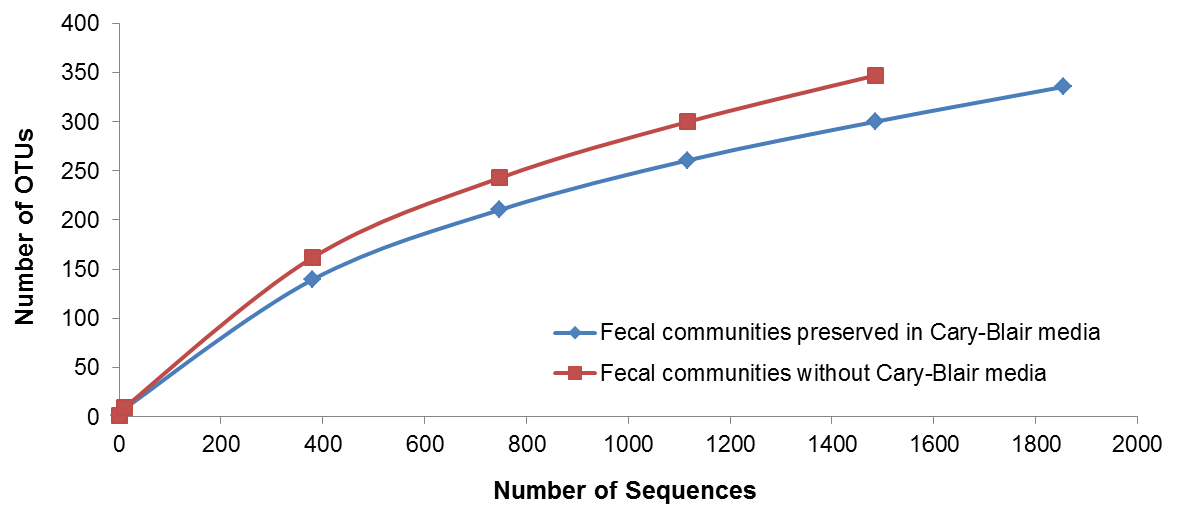


**Figure S10**. Rarefaction analysis representing the number of operational taxonomic units (OTUs) by the number of sequences sampled for four stools prepared using the MoBio and QiAmp DNA isolation kits.


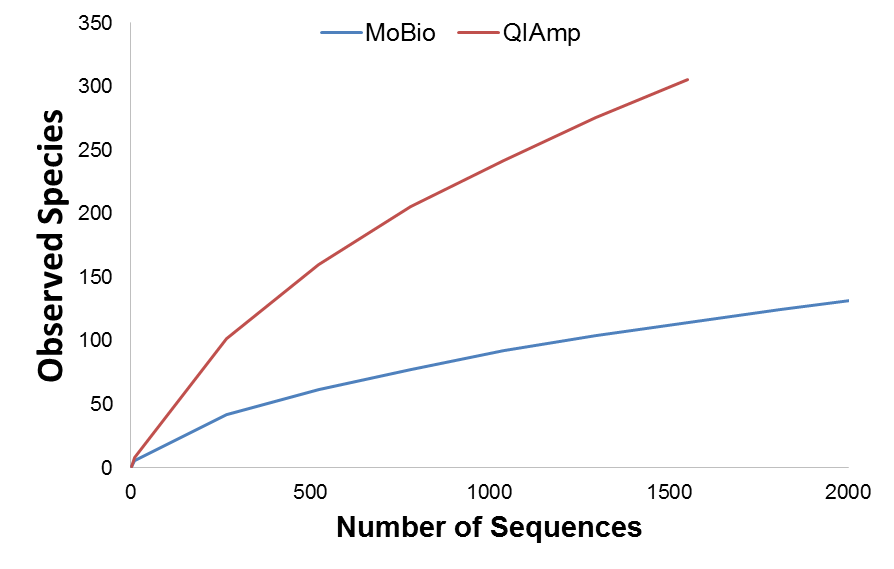


Fig. S11. Phlya abundance and composition among DNA from five stool samples recovered using both the MoBio kit (_1) and the QIAmp kit (_2)
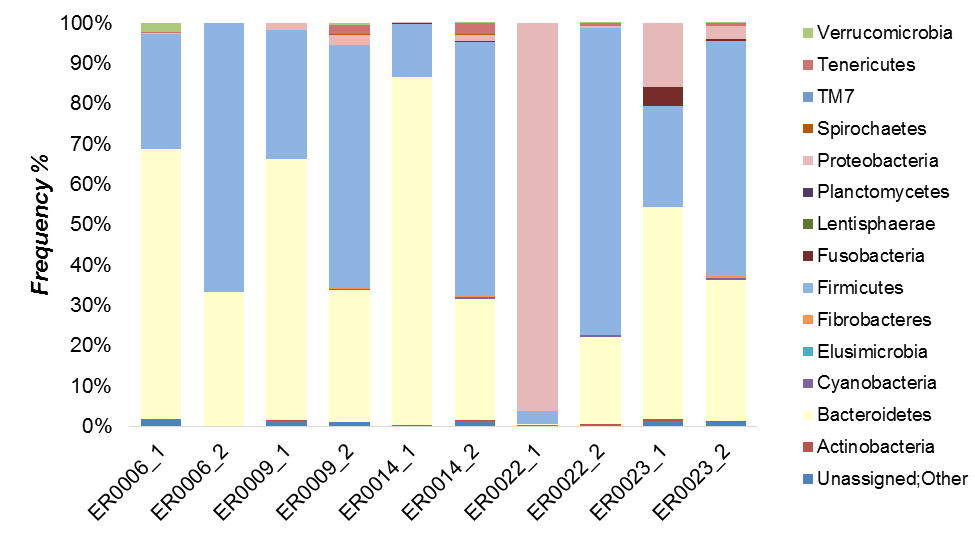
.
